# SARS-CoV-2 breakthrough infection in vaccinees induces virus-specific nasal-resident CD8^+^ and CD4^+^ T cells of broad specificity

**DOI:** 10.1084/jem.20220780

**Published:** 2022-08-16

**Authors:** Joey Ming Er Lim, Anthony Tanoto Tan, Nina Le Bert, Shou Kit Hang, Jenny Guek Hong Low, Antonio Bertoletti

**Affiliations:** 1 Programme in Emerging Infectious Diseases, Duke-NUS Medical School, Singapore; 2 Department of Infectious Disease, Singapore General Hospital, Singapore

## Abstract

Rapid recognition of SARS-CoV-2–infected cells by resident T cells in the upper airway might provide an important layer of protection against COVID-19. Whether parenteral SARS-CoV-2 vaccination or infection induces nasal-resident T cells specific for distinct SARS-CoV-2 proteins is unknown. We isolated T cells from the nasal mucosa of COVID-19 vaccinees who either experienced SARS-CoV-2 infection after vaccination (*n* = 34) or not (*n* = 16) and analyzed their phenotype, SARS-CoV-2 specificity, function, and persistence. Nasal-resident SARS-CoV-2–specific CD8^+^ and CD4^+^ T cells were detected almost exclusively in vaccinees who experienced SARS-CoV-2 breakthrough infection. Importantly, the Spike-specific T cells primed by vaccination did not suppress the induction of T cells specific for other SARS-CoV-2 proteins. The nasal-resident T cell responses persisted for ≥140 d, with minimal sign of waning. These data highlight the importance of viral nasal challenge in the formation of SARS-CoV-2–specific antiviral immunity at the site of primary infection and further define the immunological features of SARS-CoV-2 hybrid immunity.

## Teaser

Virus-specific T cells in the nasal cavity might provide an immediate layer of protection against SARS-CoV-2. This study detected SARS-CoV-2–specific nasal-resident T cells in vaccinees only after infection, highlighting the significance of nasal challenge in the formation of antiviral immunity at the site of infection.

## Graphical Abstract

**Figure:**
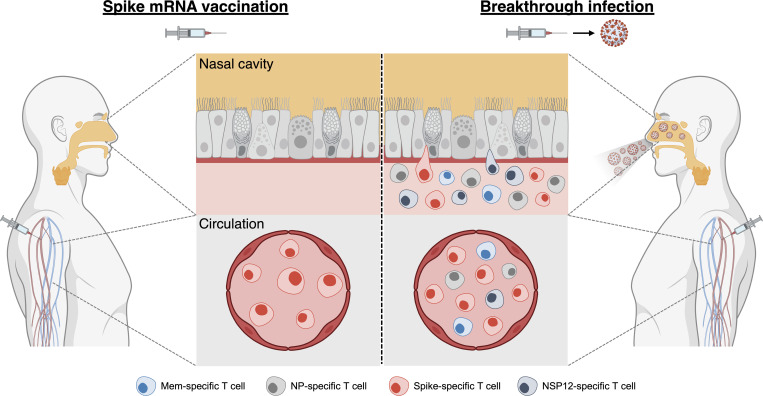

## Introduction

The human upper respiratory tract is the point of entry and the site of initial SARS-CoV-2 replication (Ahn et al., 2021). Nasal ciliated cells are easily infected due to their high ACE-2 receptor expression and sustain the bulk of initial virus production in vivo (Ahn et al., 2021). In vitro studies have shown that nasal epithelial cells can maintain high levels of viral replication for several weeks despite activation of IFN-α–mediated genes (Gamage et al., 2022), since SARS-CoV-2 can disrupt multiple intracellular antiviral immunity pathways (Banerjee et al., 2020) and block the antiviral efficacy of IFN-α (Xia et al., 2020). Resident T cells that quickly recognize virus-producing infected cells in the nasal cavity can play an important role in rapidly containing and eliminating SARS-CoV-2 infection (Ishii et al., 2022; Zhao et al., 2016), especially after the emergence of Omicron variants of concern that elude the preventive efficacy of the neutralizing antibodies induced by vaccination (Cao et al., 2021).

In animal models of mucosal virus infection, virus-specific tissue-resident CD8^+^ T cells act as a first layer of protection. They recognize the virus-infected cells and activate innate and adaptive immunity (Schenkel et al., 2014). In infection with respiratory viruses, such as influenza or respiratory syncytial virus, the presence or the adoptive transfer of tissue-resident CD8^+^ T cells in the nasal cavity controls viral spread and disease severity (Pizzolla et al., 2017a; Kinnear et al., 2018). In animal models of coronavirus infection, protection was dependent on induction of CD4^+^ T cells in the upper airway (Zhao et al., 2016). These airway-resident coronavirus-specific CD4^+^ T cells recruited coronavirus-specific CD8^+^ T cells by IFN production (Zhao et al., 2016).

Recent data in mice treated with different vaccine formulations eliciting SARS-CoV-2–specific CD8^+^ or CD4^+^ T cells in the upper airway further confirmed the importance of localized mucosal immunity in SARS-CoV-2 control (Mao et al., 2022 *Preprint*; Ishii et al., 2022). However, our knowledge of SARS-CoV-2 T cells in vaccinated or infected individuals has mainly been focused on the analysis of peripheral blood, with only few remarkable exceptions.

SARS-CoV-2–specific tissue-resident T cells have been observed in human lymph nodes and multiple organs (particularly in the lungs) in SARS-CoV-2–infected individuals (Grau-Expósito et al., 2021; Poon et al., 2021), and an analysis of the TCR repertoire of T cells purified from the upper airway of four SARS-CoV-2–infected individuals showed a persistence of Spike-specific CD8^+^ T cells in the nasal cavity ≤2 mo after infection (Roukens et al., 2022).

T cells specific for SARS-CoV-2 peptides, likely induced by seasonal coronaviruses, have also been detected in the lymphoid tissue of the oral cavity (Niessl et al., 2021) and bronchoalveolar lavage (BAL; Maini et al., 2022 *Preprint*) of healthy individuals.

Finally, while a recent study reported the presence of a very high frequency of Spike-specific T cells (≤10–20% of total nasal T cells) in the airway of mRNA vaccinated individuals (Ssemaganda et al., 2022), others detected Spike-specific T cells only in BAL of SARS-CoV-2–infected convalescents and not in BAL of vaccinated-only individuals (Tang et al., 2022). Thus, SARS-CoV-2–specific tissue-resident T cells were detected in the upper and lower airway of infected or healthy individuals, but the impact of parenteral vaccination or infection on the breadth and magnitude of SARS-CoV-2–specific T cells needs clarifications. Thus, our objective was to evaluate the presence of nasal-resident SARS-CoV-2–specific T cells and their functionality and persistence in vaccinated donors who have or have not experienced SARS-CoV-2 breakthrough infection.

## Results and discussion

### Characteristics of vaccinated individuals with and without breakthrough infection and collection of nasal cells

We studied the phenotype and SARS-CoV-2 antigen specificities of lymphocytes obtained from the nasal secretion of individuals who were either only vaccinated with BNT162b2 (naive vaccinees) or who experienced a breakthrough infection with Omicron after two or three doses of BNT162b2 (convalescent vaccinees).

**Figure 1.**
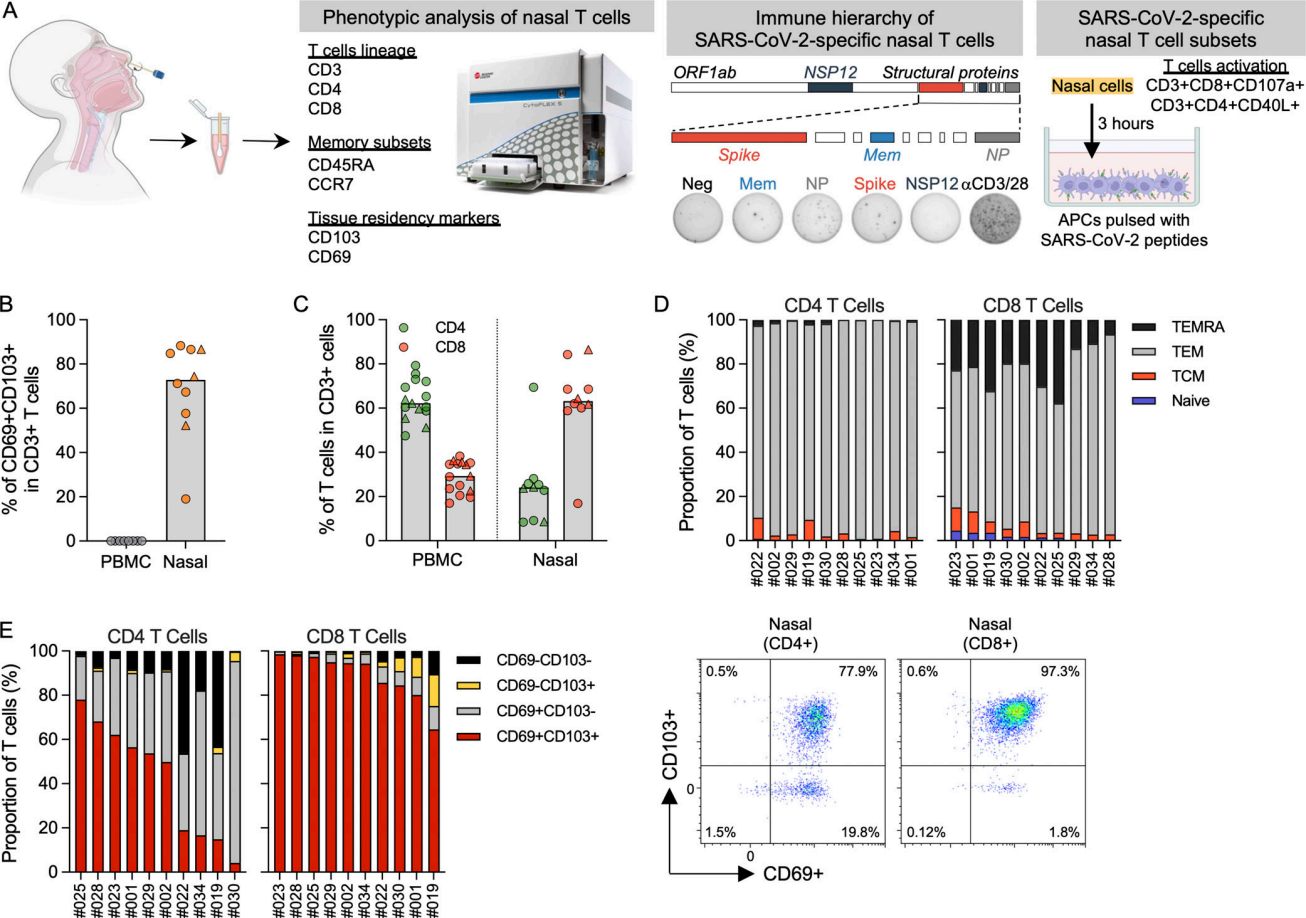
**Phenotypic analysis of T cells in nasal secretion. (A)** Schematic of experimental design. **(B)** Frequency of tissue-resident T cells present in PBMCs (*n* = 8) and nasal secretion (*n* = 10). Convalescent vaccinees are indicated by a triangle symbol. **(C)** Frequency of CD4 and CD8 T cells present in PBMCs (*n* = 14) or nasal cells (*n* = 10). Convalescent vaccinees are indicated by a triangle. **(D)** Proportion of naive (CCR7^+^CD45RA^+^), central (CCR7^+^CD45RA^−^; TCM), effector (CCR7^−^CD45RA^−^; TEM) and terminally differentiated (CCR7^−^CD45RA^+^; TEMRA) memory CD4^+^ and CD8^+^ nasal T cells (*n* = 10). **(E)** Proportion of tissue-resident marker (CD69 and CD103) expression on CD8 and CD4 nasal T cells (*n* = 10) and corresponding representative plots.

Table 1 summarizes the epidemiological characteristics of both groups and the time of nasal sample collection in relation to the last vaccination or infection. Nasal lining fluids were collected using flocked swabs introduced into the inferior turbinate, a method that is well tolerated and does not cause microlesions of nasal mucosa that could result in blood contamination of the specimens (Jochems et al., 2017). The collected swabs were placed in 1 ml cell culture medium with 1 mM dithiothreitol (DTT) and vigorously washed to dislodge the cells. The cells were then used for phenotypic analysis and different T cell assays (Fig. 1 A).

**Table 1.** Summarized demographics and vaccination status of donors analyzed

Characteristic	Naive vaccinees	Convalescent vaccinees
Number of donors	16	34
Sex	7 female, 9 male	20 female, 14 male
Age (yr)	32.6 (24–64)	35.0 (20–63)
Time since recovery (d)	Not applicable	74.6 (7–180)
Nasal lymphocytes retrieved (*n*)	5.4 × 10^4^ (3.2–9.7 × 10^4^)	6.0 × 10^4^ (3.2–11.0 × 10^4^)
**Vaccination history**		
Number of doses (mRNA vaccine)	16 three doses	18 two doses; 16 three doses
Time since last vaccine dose (d)	57.4 (8–149)	189.2 (11–440)

Numbers in parentheses indicate the range of the data set.

### Phenotype of localized nasal T cells

**Figure S1.**
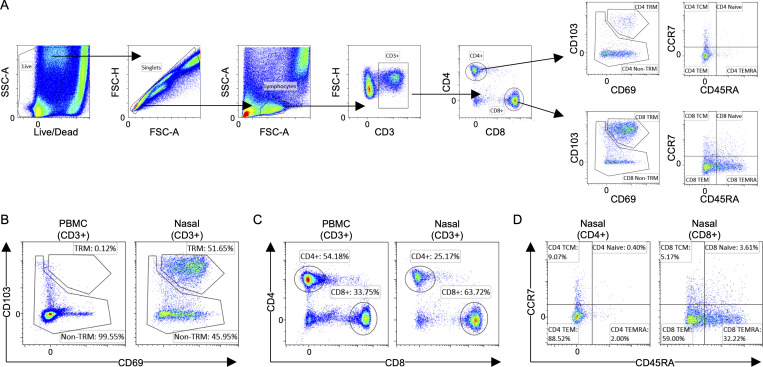
**Flow cytometric analysis of nasal T cells. (A)** Representative gating strategy of phenotypic analysis of nasal T cells. FSC, forward scatter; SSC, side scatter. **(B)** Representative flow plots of expression of tissue-residency markers on PBMCs and nasal cells. **(C)** Representative flow plots showing the proportion of CD4 and CD8 T cells present in PBMCs and nasal cells. **(D)** Representative flow plots showing the proportion of memory subsets in CD4 and CD8 nasal T cells.

We first defined the quantity of lymphocytes expressing markers of T cell lineage (CD3), tissue residency (CD69 and CD103), memory subsets (CCR7 and CD45RA), and helper (CD4) or cytotoxic (CD8) T cells in 10 individuals, 3 of whom were convalescent vaccinees (Fig. S1 A). This phenotypic analysis demonstrated that CD3^+^ lymphocytes can be collected from the nasal secretion and that the majority of nasal CD3^+^ lymphocytes expressed tissue-resident markers (Figs. 1 B and S1 B). Subtyping of the nasal CD3^+^ lymphocytes showed a predominant CD8^+^ T cell population, unlike what we observed in the blood, where CD4^+^ T cells were the more dominant population (Figs. 1 C and S1 C). We also analyzed the expression of CD45RA and CCR7: the majority of the T cells were effector memory T cells (TEM; CD4^+^, <90%; CD8^+^, <60%) or terminally differentiated memory T cells (TEMRA; CD4^+^, <5%; CD8^+^, <20%), while naive T cells were present at an extremely low frequency (Figs. 1 D and S1 D). Tissue-resident CD4^+^ and CD8^+^ T cells expressed both CD69 and CD103. Although CD103 expression on CD4^+^ tissue-resident T cells was less clear, a substantial population of CD103^+^ tissue-resident CD4^+^ T cells have been detected in the lung (Kumar et al., 2017). More importantly, the majority (∼90%) of CD8^+^ T cells expressed tissue-residency markers (CD69^+^CD103^+^), while only ∼40% of the CD4^+^ T cells were CD69^+^CD103^+^ (Fig. 1 E). There were no observable differences in the phenotype and frequency of nasal lymphocytes between the convalescent and naive vaccinees.

### Characterization of SARS-CoV-2 specificity of nasal-resident T cells

**Figure 2.**
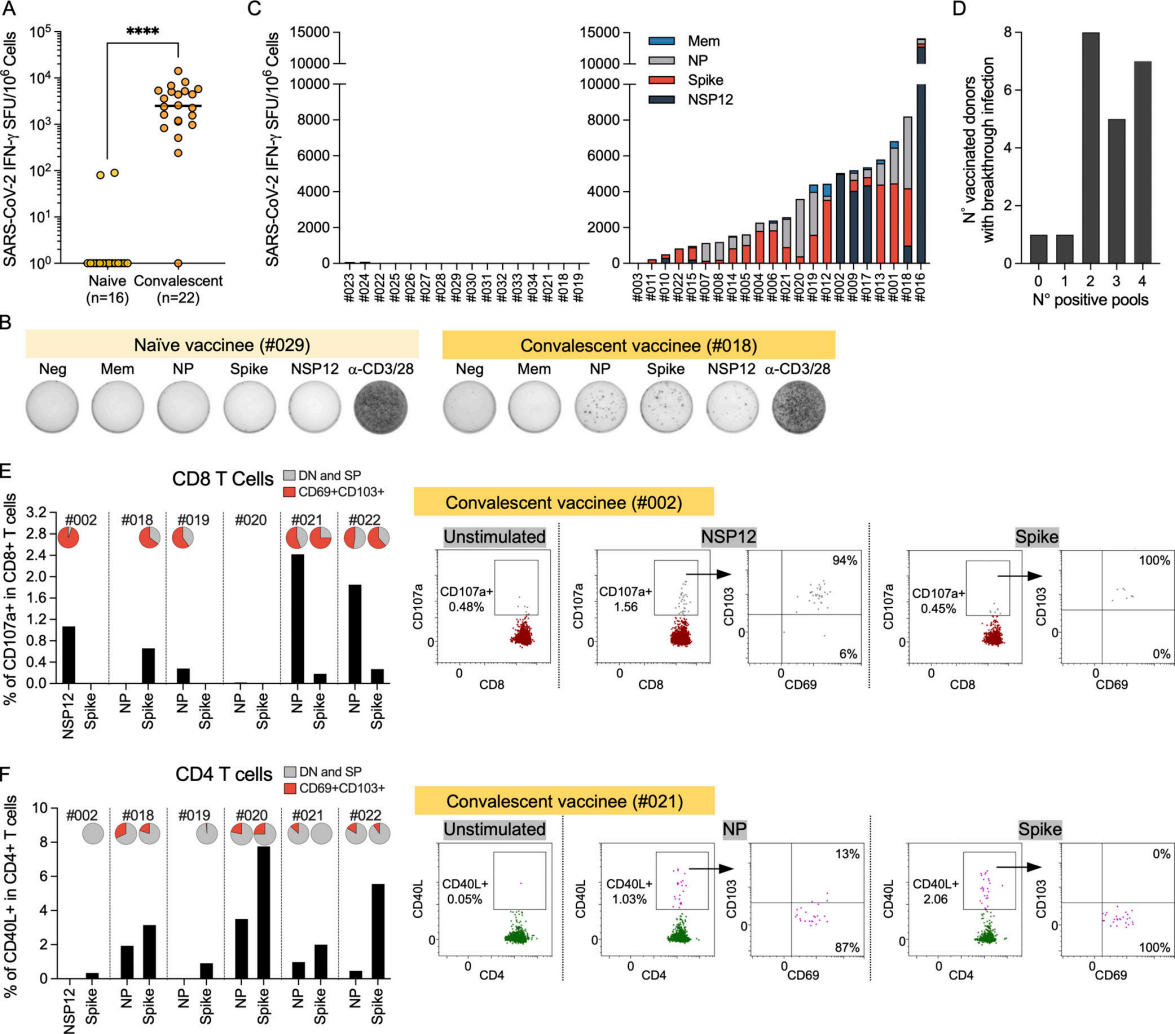
**Detection of SARS-CoV-2 nasal-resident T cells in convalescent vaccinees but not naive vaccinees. (A)** Total frequency of SARS-CoV-2–specific T cell responses in vaccinated without (naive; *n* = 16) and with (2–8 wk after recovery, convalescent; *n* = 22) breakthrough infection. Lines denote the median value of each group. Each dot represents an individual. Mann–Whitney *U* test was performed for statistical analysis. ****, P < 0.0001. **(B)** Representative ELISpot wells of nasal cells activated with SARS-CoV-2 peptide pools from vaccinees without (#029) and with (#018) breakthrough infection. **(C)** Stacked bars represent the magnitude of SARS-CoV-2–specific T cell responses targeting different peptide pools in vaccinated without (left; *n* = 16) and with (2–8 wk after recovery; right; *n* = 22) breakthrough infection. Different colored bars represent response to indicated SARS-CoV-2 peptide pools. **(D)** Bar graph shows the number of vaccinated with breakthrough infection (*n* = 22) responding to the indicated quantity of SARS-CoV-2 peptide pools. **(E and F)** Frequency of CD107a^+^ CD8 nasal T cells (E) and CD40L^+^CD4 nasal T cells (F) detected in vaccinated donors (*n* = 6) who experienced breakthrough infection, tested 2–12 wk after recovery. Pie charts represent the proportion of CD69^+^CD103^+^ and DN (double negative; CD69^−^CD103^−^) and SP (single positive; CD69^−^CD103 and CD69^+^CD103) antigen-specific T cells. Corresponding representative dot plots of frequency of CD107a^+^CD8^+^ nasal T cells and CD40L^+^CD4^+^ nasal T cells after stimulation with SARS-CoV-2–specific peptide pools are shown on the right. The background was subtracted based on the unstimulated control for each donor.

**Figure S2.**
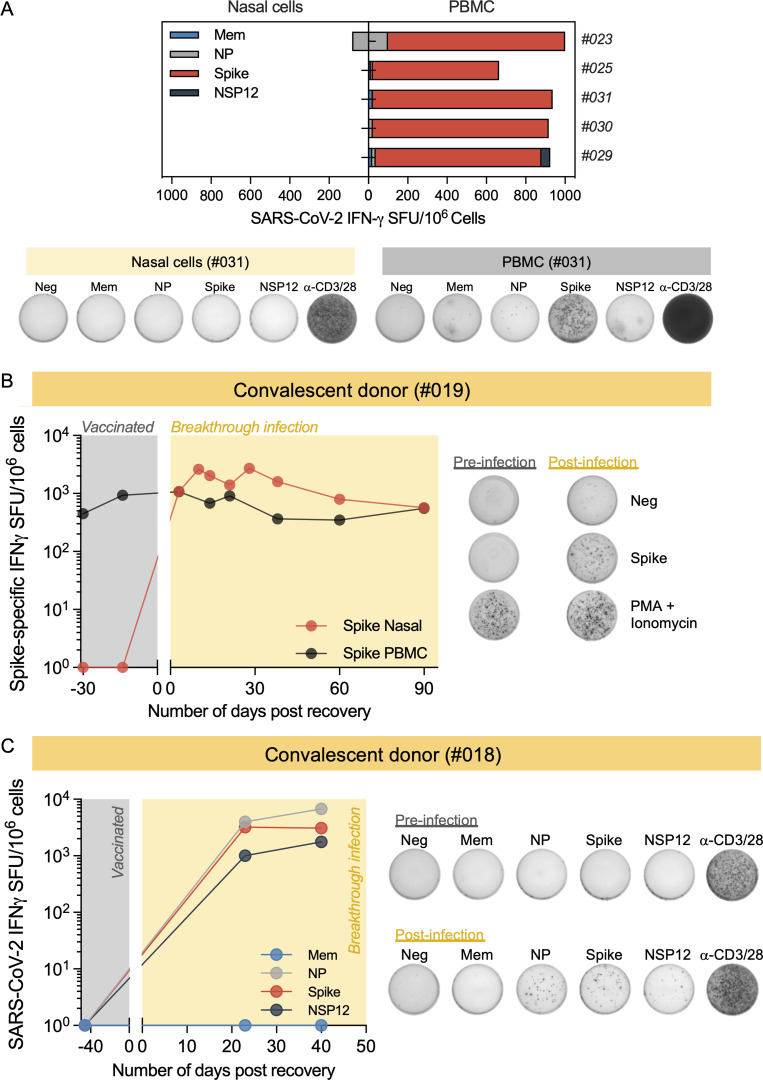
**Representative ELISpot wells showing SARS-CoV-2–specific T cells in naive and convalescent vaccinees. (A)** Paired analysis of IFN-γ SFU frequency in nasal cells and PBMCs stimulated with SARS-CoV-2 peptide pools of naive vaccinees (*n* = 5). Below, representative ELISpot wells from nasal cells and PBMCs. **(B)** Longitudinal analysis of Spike-specific IFN-γ SFU frequency present in nasal cells and PBMCs of a vaccinated individual (#019) before and after breakthrough infection. Right, representative ELISpot wells of Spike-specific T cells of vaccinated individual (#019) before and after breakthrough infection. **(C)** Longitudinal analysis of SARS-CoV-2–specific IFN-γ SFU frequency present in nasal cells of a vaccinated individual (#018) before and after breakthrough infection. Responses to peptide pools covering different SARS-CoV-2 proteins are indicated by color coding. Right, representative ELISpot wells of nasal T cell response from vaccinated donor before and after breakthrough infection.

We then analyzed SARS-CoV-2 specificity of T cells collected from the nasal cavity. The cells were activated with peptide pools covering structural proteins (membrane [Mem], nucleoprotein [NP], and Spike), which induce T cell responses in mild/asymptomatic SARS-CoV-2 convalescents (Le Bert et al., 2021), and nonstructural protein 12 (NSP12), which induced T cells in individuals with abortive infection (Swadling et al., 2021). We derived the estimated quantity of lymphocytes in each sample by flow cytometry analysis, and we diluted nasal collected cells to perform ELISpot assays with ≥5,000 lymphocytes/well. Positive controls were obtained by stimulating cells with anti-CD3/CD28 beads, and negative controls consisted of unstimulated cells. Data were analyzed only if positive controls showed ≥100 spots. Remarkably, SARS-CoV-2 peptide pools did not elicit any ELISpot response in the 16 naive vaccinees (14 individuals were negative, and 2 individuals had 1–2 spots), despite the presence of Spike-specific T cells in circulation (Fig. 2, A and B; and Fig. S2 A). In contrast, SARS-CoV-2 peptide pools activated IFN-γ production by nasal cells in almost all convalescent vaccinees (19 of 22 showed ≥10 spots in each well seeded with 5,000–10,000 lymphocytes; frequency indicated out of 1 × 10^6^ normalized T cells; Fig. 2 A). The requirement of infection for detection of SARS-CoV-2–specific nasal T cells was further supported by data of two vaccinated individuals who were analyzed before and after breakthrough infection. In both donors, SARS-CoV-2–specific T cells were detected in the nasal samples only after breakthrough infection (Fig. S2, B and C). Of note, in donor #019 (Fig. S2 B), we traced longitudinally Spike-specific T cells both in blood and nose before and after infection and observed that the detection of Spike-specific T cells in the nasal cavity after infection was not associated with an increased frequency of Spike-specific T cells in the blood.

The response of nasal cells to SARS-CoV-2 peptide pools was heterogeneous in convalescent vaccinees, targeting not only Spike, but also other structural and/or nonstructural proteins (Fig. 2 C). Spike peptide pool triggered IFN-γ spot formation in 19 of 22 tested individuals but was dominant in only 9 of 22. In five individuals, nasal lymphocytes recognized preferentially the NP peptide pool, while an NSP-12–specific nasal T cell response was dominant in four individuals. In most of the convalescent vaccinees, nasal T cells recognized two to four different proteins (Fig. 2 D).

We then characterized in selected convalescent vaccinees whether nasal SARS-CoV-2–specific T cells were CD4^+^ or CD8^+^. Nasal samples were incubated for 3 h with autologous circulating monocytes pulsed or not with peptide pools (Fig. 1 A). After incubation, we evaluated by flow cytometry the CD3^+^CD8^+^ or CD3^+^CD4^+^ cells with induced expression of CD107a or CD40L, respectively. Background determined by unstimulated nasal cells was subtracted for each donor. Fig. 2, E and F, show that antigen-specific nasal-resident T cells could be either CD8^+^ or CD4^+^ T cells. Degranulating CD107a^+^CD8^+^ T cells were present in five of six convalescent vaccinees who donated additional samples for this analysis, and the majority of the CD107a^+^CD8^+^ T cells were nasal-resident (CD69^+^CD103^+^) T cells. Interestingly, the strongest responses that were detected in three convalescent donors were targeting non-Spike peptide pools, NP and NSP12. These features can provide better recognition of Omicron variants (Marking et al., 2022 *Preprint*), since the NSP-12– and NP-specific CD8^+^ T cells are less likely to be affected by the amino acid mutations preferentially present in the Spike protein of Omicron BA.1 and BA.2. Nasal-resident CD40L^+^CD4^+^ T cells were detected in five of six individuals, and they were made up of mostly CD69^+^CD103^−^ T cells, which is dissimilar to the antigen-specific nasal-resident CD8^+^ T cells.

### Comparison of SARS-CoV-2–specific T cells in blood and nasal mucosa

**Figure 3.**
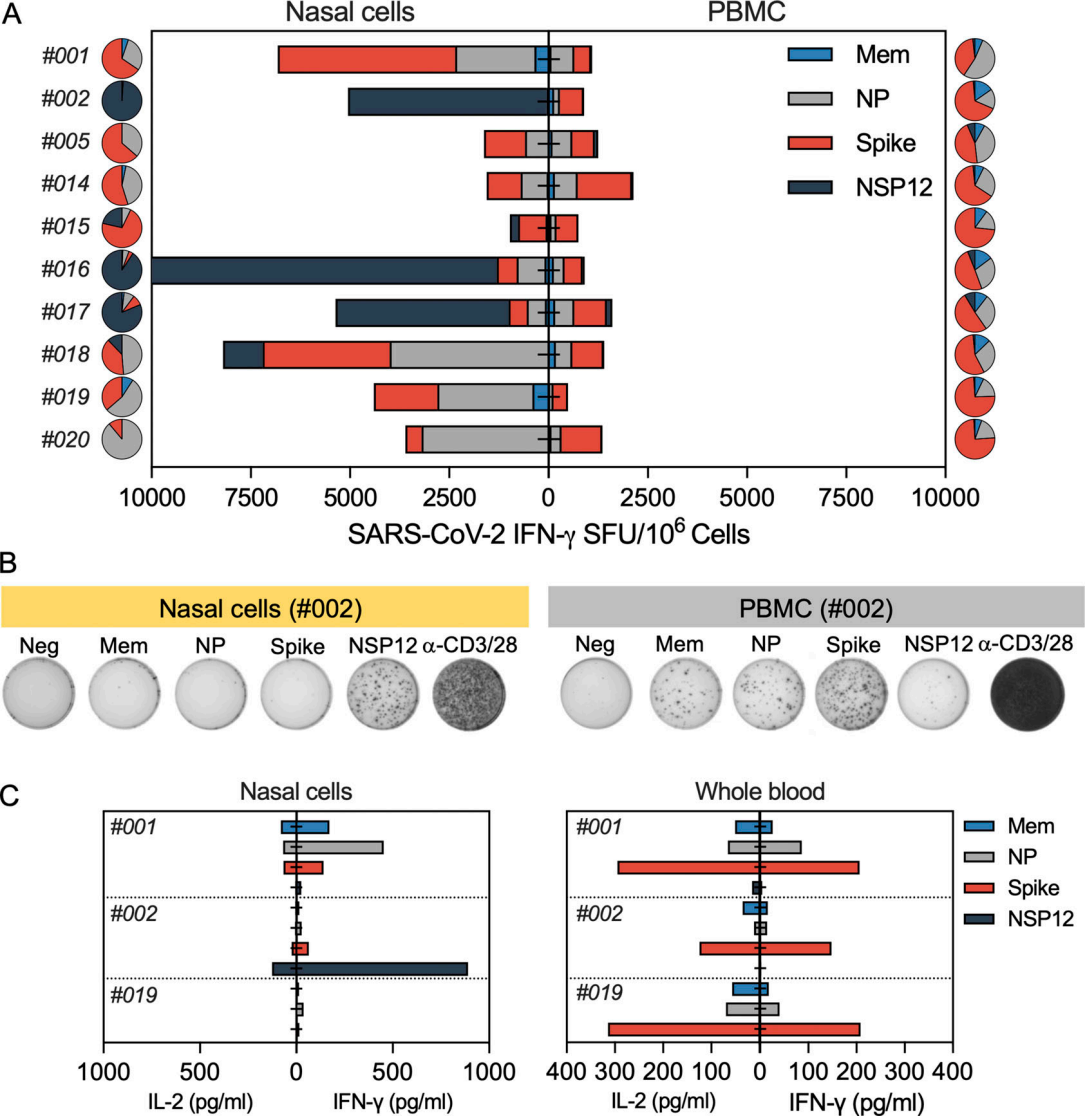
**SARS-CoV-2–specific response of nasal and circulating T cells.** **(A)** Paired analysis of IFN-γ SFU frequency in nasal cells (left) and PBMCs (right) stimulated with SARS-CoV-2 peptide pools of vaccinated with breakthrough infection (*n* = 10) tested 1–2 mo after recovery. Pie charts represent the percentage of IFN-γ SFU reactive to the individual SARS-CoV-2 peptides pools; **(B)** Representative ELISpot wells from nasal cells and PBMCs. **(C)** Paired analysis of cytokines (IFN-γ and IL-2) secreted in whole blood (320 μl whole blood and 80 μl medium; unnormalized) and nasal cells (30 μl of medium; normalized to 100,000 lymphocytes/well) after stimulation with different SARS-CoV-2 peptide pools.

We analyzed in parallel the ability of nasal cells and peripheral blood mononuclear cells (PBMCs) to recognize the distinct peptide pools (Mem, NP, Spike, and NSP12) after breakthrough infection. Fig. 3 A shows that the profile of antigen recognition by nasal and circulating T cells was different. As expected, circulating SARS-CoV-2–specific T cells always include a large proportion of Spike-specific T cells. Spike T cell responses in the peripheral blood were dominant in 9 of 10 convalescent vaccinees, but such dominance was not always detected in T cells collected from the nasal mucosa. Spike-specific nasal-resident T cells were dominant in only 4 of 10 tested convalescent vaccinees, while NSP-12–specific nasal-resident T cells were dominant in three convalescent vaccinees and NP-specific nasal-resident T cells in another three convalescent vaccinees. Thus, infection causes a hierarchy of SARS-CoV-2–specific T cell responses that is independent of the dominance of vaccine-induced Spike-specific T cells in the blood (Fig. 3, A and B).

**Figure S3.**
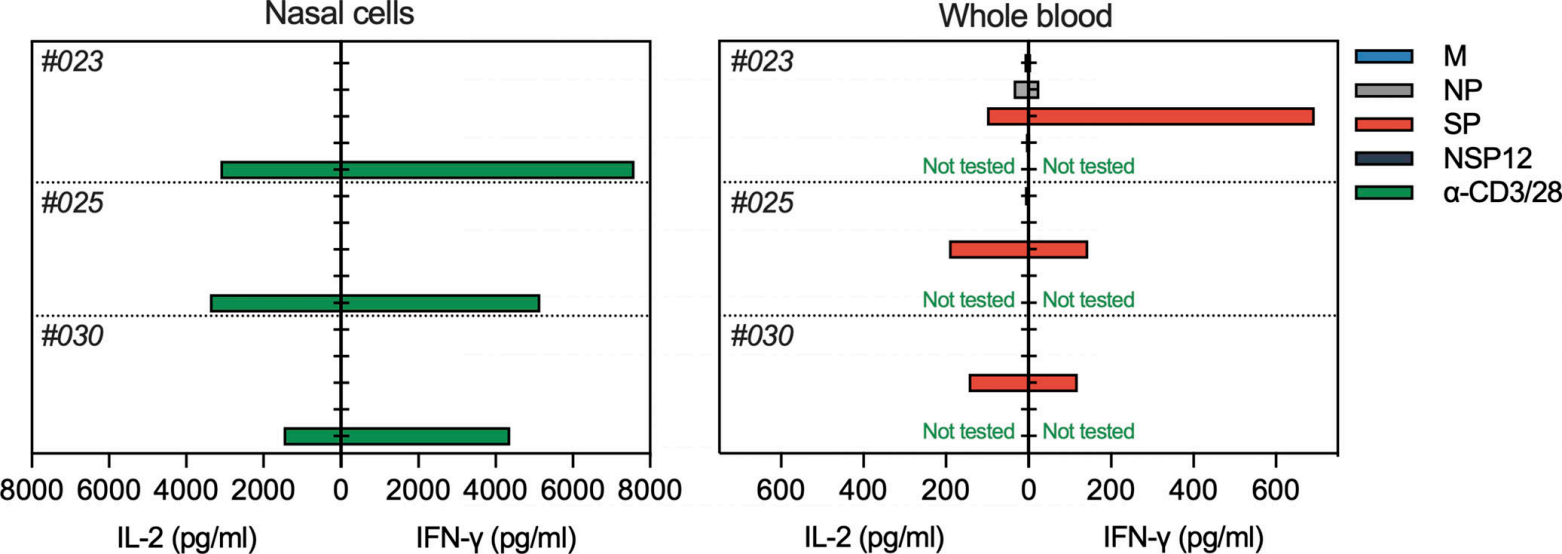
Paired analysis of cytokines (IFN-γ and IL-2) secreted by nasal cells and whole blood of vaccinated donors (*n* = 3) after overnight stimulation with different SARS-CoV-2 peptide pools.

We also compared the ability of nasal and circulating T cells to produce IFN-γ and IL-2 cytokines after specific peptide stimulation. Nasal cells from convalescent and naive vaccinees were stimulated with SARS-CoV-2 peptide pools in 30 μl medium, and supernatants were collected the next day to analyze the levels of secreted IFN-γ and IL-2. In convalescent vaccinees, an exclusive production of IFN-γ was detected only after peptide pool stimulation, yet IL-2 was barely detectable (Fig. 3 C). This profile was different from the cytokine secretion profile of whole blood (320 μl), in which high levels of both IFN-γ and IL-2 were present after peptide stimulation. In naive vaccinees, no cytokines were detected after peptide stimulation of the nasal cells (Fig. S3).

### Persistence of SARS-CoV-2–specific T cells in the nasal mucosa

**Figure 4.**
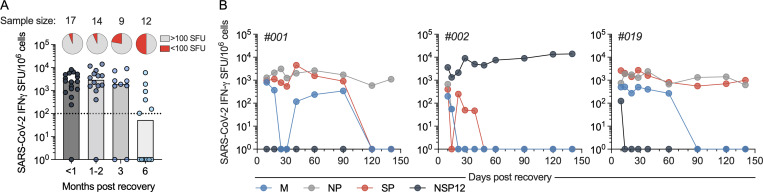
**Persistent presence of SARS-CoV-2–specific nasal-resident T cells for ≤140 d. (A)** Cross-sectional analysis of total SARS-CoV-2–specific T cell responses (Mem, NP, NSP-12, and Spike combined) from vaccinated donors after breakthrough infection at different months after recovery. The pie charts represent the proportion of subjects who had SARS-CoV-2–specific T cell responses higher or lower than 100 SFU/10^6^ lymphocytes. Each dot represents a sample. The corresponding sample size of each group are stated at the top. **(B)** Longitudinal analysis of IFN-γ SFU frequency for ≤140 d in nasal cells (*n* = 3). Responses to peptide pools covering different SARS-CoV-2 proteins are indicated by color coding.

We analyzed convalescent vaccinees at different time points (<1, 1–3, and 6 mo) after recovery of the infection and cross-sectionally evaluated the persistence of SARS-CoV-2–specific T cells. For ≤3 mo, most of the convalescent vaccinees had detectable SARS-CoV-2–specific T cell responses that remained consistent for the duration, with no sign of waning (Fig. 4 A). At 6 mo, half of the donors (6 of 12) did not have detectable SARS-CoV-2–specific T cells, showing that nasal-resident T cell responses waned only 3 mo after recovery.

Additionally, we longitudinally evaluated the maintenance of SARS-CoV-2–specific T cells in the nasal cavity in three convalescent vaccinees. Nasal samples were collected at multiple time points for 140 d after recovery from infection and analyzed for the presence of SARS-CoV-2–specific T cells. We detected SARS-CoV-2–specific T cell responses at all time points after recovery from infection (Fig. 4 B). Specifically, all three donors had T cell responses against some specific SARS-CoV-2 peptide pools that persisted with no sign of frequency reduction, while we also observed waning of some responses at around 90–120 d (Fig. 4 B). The reduction of responses to certain peptide pools at 90–120 d was consistent with the cross-sectional analysis demonstrating that at 6 mo after recovery, in those donors with detectable responses, the number of nasal-resident SARS-CoV-2–specific T cells was lower. These data support the concept of long-term persistence of nasal-resident T cells (≤140 d), with potential waning of T cell responses 3–4 mo after recovery, in line with data in an animal model of influenza infection (Pizzolla et al., 2017a). Further longitudinal analysis will be necessary to test whether such SARS-CoV-2 nasal-resident T cells can persist for years, as we have seen in circulating memory T cells after SARS-CoV-1 infection (Le Bert et al., 2020).

Taken together, we demonstrated that SARS-CoV-2 breakthrough infections of vaccinees led to the detection of tissue-resident CD4^+^ and CD8^+^ T cells specific for different SARS-CoV-2 proteins in the upper airway. These SARS-CoV-2 multispecific tissue-resident T cells persisted at levels detectable with assays measuring T cell functionality (IFN-γ production by ELISpot, cytokine release assay, and AIM assay with T cell degranulation marker) for ≥140 d after infection. In contrast, identical assays were unable to detect any T cells specific for Spike or any other SARS-CoV-2 proteins in the upper airway of almost all vaccinated only individuals despite the presence of Spike-specific T cells in their peripheral blood. Even though we cannot exclude that some vaccine-induced Spike-specific T cells might be present in the upper airway immediately after vaccination boost or be present in our samples at frequencies lower than the detection limit of our assay (ELISpot assay were performed with ∼5–10 × 10^3^ T cells), our results do not confirm the extremely high frequency of Spike-specific CD8^+^ T cells recently reported in the nasal cavity 2 and 6 mo after vaccination (Ssemaganda et al., 2022). Instead, our results are in line with the inability to detect a robust Spike-specific T cell response in the BAL of naive vaccine recipients (Tang et al., 2022). It will be important for other groups to try to resolve this profound discrepancy between the reported results, using an array of different assays for T cell detection. Thus, SARS-CoV-2 infection and not vaccination alone recruits a robust population of T cells specific for different SARS-CoV-2 proteins into the nasal mucosa. This is in line with the observations of the presence of Spike-specific T cells in the nasal mucosa and BAL of COVID-19 convalescent patients (Roukens et al., 2022; Tang et al., 2022). This is also compatible with the data in animal models of influenza infection in which virus-specific CD8^+^ T cells were recruited to the upper airway by increased antigen deposition in loco and by “antigen independent” mechanisms of inflammation mediated by the local infection (Pizzolla et al., 2017a).

Our data do not clarify whether SARS-CoV-2–specific T cells detected in the nasal mucosa of vaccinees after infection were primed in nasal-associated lymphoid tissue or in more organized lymphoid structures present in the upper airway (pharyngeal, lingual, and palatine tonsils). Data in animal models suggest that nasal-associated lymphoid tissues do not prime virus-specific T cells but support their persistence (Pizzolla et al., 2017b). Nevertheless, our inability to detect SARS-CoV-2–specific cross-reactive T cells in the nasal secretion of vaccinated only individuals was unexpected, because cross-reactive SARS-CoV-2–specific T cells have been detected in the pharyngeal tonsils and BAL of healthy unvaccinated individuals (Maini et al., 2022 *Preprint*; Niessl et al., 2021). For example, a clear enrichment was reported of SARS-CoV-2 T cells in BAL compared with blood, with 0.5–1% of CD4^+^ T cells and 0.1–0.4% of CD8^+^ T cells, and these frequencies are numerically compatible with the detection limit of our IFN-γ ELISpot assays. However, the nasal cavity has different characteristics of induction, recruitment, and persistence of T cells compared with the lung (Pizzolla et al., 2017a), which could explain the differences in the findings. An alternative interpretation of our ability to exclusively detect SARS-CoV-2 T cells in the nasal mucosa of infected vaccinated individuals might be due to the possible differences in T cell affinity of nasal-resident T cells and cross-reactive SARS-CoV-2 T cells detected in the tonsils and BAL of healthy individuals (Maini et al., 2022 *Preprint*; Niessl et al., 2021). SARS-CoV-2 cross-reactive T cells can have lower affinity to SARS-CoV-2 epitopes (Bacher et al., 2020) and have been shown to produce lower amounts of IFN-γ (Maini et al., 2022 *Preprint*; Niessl et al., 2021).

We characterized the presence of SARS-CoV-2 T cells by direct stimulation of nasal T cells with peptide pools (without using costimulation with α-CD28 antibodies). Our method might detect preferentially high affinity SARS-CoV-2–specific T cells that produce a substantial amount of IFN-γ. The cytokine release assays of nasal and circulatory SARS-CoV-2–specific T cells showed that nasal-resident T cells secrete high quantities of IFN-γ. The detailed analyses of TCR affinity and transcriptomic profiles of SARS-CoV-2–specific T cells resident in different tissues is needed to define these possibilities.

Irrespective of the causes of undetectable Spike-specific T cells in the nasal mucosa of individuals who were only vaccinated, SARS-CoV-2 breakthrough infection in vaccinees clearly triggered a specific accumulation of highly functional SARS-CoV-2 CD8^+^ and CD4^+^ T cells in the nasal mucosa, with a hierarchy of protein recognition that was different to what was detected in the circulation. These multi-SARS-CoV-2 protein-specific T cells might quickly trigger a localized secretion of the antiviral IFN-γ cytokine after antigen encounter and start a chain of innate and adaptive immune events able to control viral replication, as shown in different mice models of viral infection (Zhao et al., 2016; Pizzolla et al., 2017a; Schenkel et al., 2014; Mao et al., 2022 *Preprint*; Kinnear et al., 2018; Ishii et al., 2022).

Despite the limited sample size of this study, our analysis of cellular immunity in nasal secretion complements the growing experimental evidence showing that infection after vaccination induces a mucosal SARS-CoV-2–specific antibody response characterized by robust production of secretory Spike-specific IgA (Isho et al., 2020; Sano et al., 2021 *Preprint*; Azzi et al., 2022; Terreri et al., 2022; Collier et al., 2022; Tang et al., 2022). Our data also provide further evidence of the peculiarity of anti-SARS-CoV-2 immunity in individuals with “hybrid” immunity. The combination of SARS-CoV-2 infection and vaccination elicit stronger antibody and T cell responses in the blood (Reynolds et al., 2021), a broad repertoire of anti-Spike-specific B cells (Terreri et al., 2022; Rodda et al., 2022), and a functional profile of circulating CD4 T cells characterized by production of IFN-γ and IL-10 (Rodda et al., 2022). This T cell cytokine profile is associated with virus-respiratory protection in animal models (Zhao et al., 2016) and mild/asymptomatic SARS-CoV-2 infection in humans (Le Bert et al., 2021; Grau-Expósito et al., 2021). Breakthrough infection has also been shown to expand the CD8^+^ T cell repertoire in the circulation (Minervina et al., 2022) and our characterization of SARS-CoV-2–specific T cells in the nasal mucosa demonstrated that expansion of CD8^+^ and CD4^+^ T cells specific for different antigens is not an exclusive feature of circulating T cells in peripheral blood. Taken together, these immunological features might explain why individuals with “hybrid” immunity can control SARS-CoV-2 Omicron replication quicker than vaccinated only (Marking et al., 2022 *Preprint*) and why a superior immune protection is induced by infection over mRNA vaccination alone (Altarawneh et al., 2022).

There are limitations in this study; in addition to the small sample size, phenotypic analysis of the nasal T cells and analysis of their SARS-CoV-2 specificity was limited to few markers of tissue residency (CD69 and CD103) and memory (CD45RA and CCR7) and selected SARS-CoV-2 proteins (Mem, NP, Spike, and NSP-12). The limited number of T cells collected imposed these limitations. To better define the breadth of the nasal-resident T cell response against SARS-CoV-2, it will be necessary to analyze their ability to recognize epitopes derived from other structural proteins, nonstructural proteins, or out-of-frame open reading frames that have been shown to induce robust CD8^+^ T cell response in circulation (Weingarten-Gabbay et al., 2021). Finally, studies of the durability of the nasal-resident SARS-CoV-2 T cells >140 d are warranted to understand the long-term impact of nasal-resident T cells in SARS-CoV-2 protection.

## Materials and methods

### Study participants

This study was approved by the SingHealth Centralized Institutional Review Board (CIRB/F 2021/2014). All 50 donors studied were vaccinated with two or three doses of mRNA vaccine, and 34 of them experienced a breakthrough infection (convalescent vaccinees) identified by COVID-19 rapid lateral flow test. The remaining donors (naive vaccinees; *n* = 16) were vaccinated only and did not experience breakthrough infection. Naive vaccinees performed a COVID-19 rapid lateral test every week (October 2021 to April 2022), and their PBMCs were tested for the presence of T cell response against Spike, Mem, and NP. They showed response only to Spike and not to Mem and NP, compatible with their SARS-CoV-2 naive status. Nasal samples were collected 7–149 d after last vaccination and 7–180 d after obtaining a negative COVID-19 rapid lateral flow test. Longitudinal nasal samples were collected from nine donors. Summarized details on the participants analyzed in this study are listed in Table 1.

### Nasal sample collection

To collect the nasal lining fluid from individual donors, flocked swabs (Copan Diagnostics) were inserted into the inferior turbinate of the donors and rotated 10–20 times. The swabs were then placed into 1 ml AIM-V with 1 mM DTT (Thermo Fisher Scientific) and incubated at 37°C. After 30 min, the tube containing the swabs was vigorously vortexed to dislodge the cells. The cells were spun down for washing to remove DTT from the medium. The isolated nasal cells were then quantified using flow cytometric analysis and used for subsequent experiments.

### PBMC isolation

Peripheral blood was collected, and PBMCs from all collected blood samples were isolated by Ficoll-Paque (Cytiva) density gradient centrifugation. Isolated PBMCs were either studied directly or cryopreserved and stored in liquid nitrogen until used in the assays.

### SARS-CoV-2 protein peptide pools

Peptides of 15-mer that overlapped by 10 amino acids spanning the entire protein sequences of NP, Mem, Spike, and NSP12 of SARS-CoV-2 were synthesized (Genscript; Le Bert et al., 2020; Swadling et al., 2021). Peptides from each protein were pooled into their respective mega-pool and used for subsequent experiments.

### IFN-γ ELISpot

Freshly collected nasal cells were stimulated with peptide pools in an IFN-γ ELISpot assay. PBMCs (400,000 cells) or nasal lymphocytes (5,000–10,000 cells) were seeded per well into ELISpot plates (Millipore Sigma) precoated with human IFN-γ antibody overnight at 4°C. Next, the cells were stimulated for 18 h with the peptide pools at 1 μg/ml. The plates were then incubated with a human biotinylated IFN-γ detection antibody, followed by streptavidin–alkaline phosphatase and developed using the KPL BCIP/NBT phosphatase substrate (Seracare Life Sciences). To quantify positive peptide-specific responses, spots of the unstimulated wells were subtracted from the peptide-stimulated wells, and the results were expressed as spot-forming units (SFU) per 10^6^ cells. Results were excluded if negative control wells had >10 spots in each well seeded with 5,000–10,000 lymphocytes or if positive control wells (anti-CD3/CD28 beads) had <100 spots.

### Nasal T cell phenotyping

Cells were resuspended in PBS and stained with Zombie NIR Fixable Viability Kit to exclude dead cells in subsequent analysis. The cells were next washed in FACS buffer with 2 mM EDTA and stained with surface markers anti-CD3-BV605 (RRID: AB_2561911; BioLegend), anti-CD4-BV650 (RRID: AB_2744425; BD), anti-CD8-PE-Cy7 (RRID: AB_396852; BD), anti-CD69-AF700 (RRID: AB_493775; BioLegend), anti-CD103-APC (RRID: AB_10669816; eBioscience), anti-CD45RA-FITC (RRID: AB_395879; BD), and anti-CCR7-BV421 (RRID: AB_2728119; BD) diluted in FACS buffer for 30 min on ice. After two more washes in FACS buffer, cells were resuspended in PBS before acquisition.

### Phenotyping of SARS-CoV-2–specific T cells

CD3^+^ cells were depleted from the freshly thawed PBMCs using EasySep Positive Selection Kits II (Stemcell). Flowthroughs consisting of CD3^−^ cells were collected. The cells (5 × 10^5^) were then pulsed with 5 μg/ml of peptides or DMSO for 1 h in 37°C. After incubation, pulsed cells were washed twice before addition of autologous nasal cells together with anti-CD40L-PE (RRID: AB_314828; BioLegend) and anti-CD107a-APC (RRID: AB_1727417; BD). After 3 h of incubation at 37°C, the cells were washed in PBS stained with Zombie NIR Fixable Viability Kit to exclude dead cells in subsequent analysis. Then, they were washed in FACS buffer and stained with surface markers anti-CD3-BV605 (RRID: AB_2561911; BioLegend), anti-CD4-BV650 (RRID: AB_2744425; BD), anti-CD8-PE-Cy7 (RRID: AB_396852; BD), anti-CD69-AF700 (RRID: AB_493775; BioLegend), and anti-CD103-FITC (RRID: AB_10597744; eBioscience) diluted in FACS buffer for 30 min on ice. After two more washes in FACS buffer, cells were resuspended in PBS before acquisition with a Beckman Coulter CytoFLEX S analyzer.

### Cytokine secretion assay

Freshly isolated nasal cells quantified by flow cytometric analysis and resuspended in 30 μl culture medium (AIM-V + 2% AB serum) or freshly drawn blood (320 μl) were diluted 0.2× with RPMI and stimulated with either 1 μg/ml peptides or the equivalent concentration of DMSO vehicle. After 16 h of incubation, the culture supernatants/plasma were collected and stored at −30°C. Cytokine concentrations in the supernatants/plasma were quantified using an ELLA machine with microfluidic multiplex cartridges that measured IFN-γ and IL-2 according to the manufacturer’s protocol. The levels of cytokines present in the supernatants of DMSO controls were subtracted from the corresponding peptide pool–stimulated samples, and the values were normalized to 100,000 nasal lymphocytes per condition.

### Online supplemental material

Fig. S1 shows the flow cytometric analysis and representative flow plots of nasal T cells. Fig. S2 shows the representative ELISpot results showing SARS-CoV-2–specific nasal and circulatory T cells in naive and convalescent vaccinated individuals. Fig. S3 shows the paired analysis of cytokines (IFN-γ and IL-2) secreted by nasal cells and whole blood of vaccinated donors after overnight stimulation with different SARS-CoV-2 peptide pools.
